# Neighborhood Environment Perceptions and the Likelihood of Smoking and Alcohol Use

**DOI:** 10.3390/ijerph120100784

**Published:** 2015-01-14

**Authors:** Nattinee Jitnarin, Katie M. Heinrich, Christopher K. Haddock, Joseph Hughey, LaVerne Berkel, Walker S.C. Poston

**Affiliations:** 1 Institute for Biobehavioral Health Research, National Development and Research Institutes, Inc., 1920 143rd Street, Suite 120, Leawood, KS 66224, USA; E-Mails: haddock@ndri.org (C.K.H.); poston@ndri.org (W.S.C.P.); 2 Department of Kinesiology, Kansas State University, Manhattan, KS 66506, USA; E-Mail: KMHPhD@ksu.edu; 3 Departments of Psychology and Architecture and Urban Planning, University of Missouri-Kansas City, Kansas City, MO 64110, USA; E-Mail: hughey.joe@gmail.com; 4 School of Education, University of Missouri-Kansas City, 615 E. 52nd Street, Kansas City, MO 64110, USA; E-Mail: berkell@umkc.edu

**Keywords:** neighborhood perceptions, neighborhood environment, smoking, heavy drinking, binge drinking, alcohol use

## Abstract

Neighborhood characteristics are important correlates for a variety of health outcomes. Among several health risk behaviors, smoking and alcohol use have significant consequences. Perceptions of neighborhood problems are associated with depressive symptoms, lower physical activity, and lower quality of life. However, it is unclear which perceived aspects of neighborhoods might be related to smoking and drinking. We examined whether perceived neighborhood characteristics were associated with smoking and drinking patterns using data from US metropolitan Midwestern area adults. Participants completed surveys including sociodemographic characteristics, neighborhood perceptions, behavioral and psychological health. For men, negative perceptions of neighborhood infrastructures were significant predictors for smoking and binge drinking. Among women, no perceived environmental factors were associated with smoking or drinking. However, education was a significant negative predictor for smoking. As age increased, the likelihood of using cigarettes, heavy and binge drinking in women decreased significantly. Depression was a positive predictor for smoking and heavy drinking in men and women, respectively. These findings indicate that the perceived neighborhood infrastructure was predictive of health behaviors among men, even after adjusting for key confounders. Closer attention may need to be paid to the role of neighborhood environmental characteristics along with individual-level characteristics in influencing unhealthy behaviors.

## 1. Introduction

Neighborhood characteristics are important correlates for a variety of health outcomes and health inequalities [1,2,3]. For example, neighborhood socioeconomic status (SES) often has been associated with residents’ health behaviors and outcomes including physical activity, obesity, and mental health [3,4,5,6,7,8]. Although it is not clear how neighborhood SES influences health status, one proposed mechanism is that low SES affects the environments of individuals by creating settings that promote unhealthy behaviors [4,9]. 

Smoking and alcohol use are important health behaviors that have significant consequences. According to the recent reports from the World Health Organization [10,11], alcohol and tobacco use are the third and sixth leading global risk factors for disease and disability, respectively. Although numerous studies indicate that individual characteristics such as education, employment and/or occupational status are associated with smoking and drinking [12,13], there is growing evidence demonstrating associations between neighborhood characteristics and smoking and drinking behaviors. [5,6,7,8,14]. For example, people living in areas of high neighborhood deprivation were more likely to smoke and use alcohol compared with those living in a neighborhood with moderate deprivation [14]. 

Several factors may explain why neighborhood characteristics influence smoking and drinking prevalence. Substandard or distressed physical features of neighborhoods (e.g., poor quality housing, litter, damage, and other incivilities) and inadequate basic resources including stores, public transportation, hospitals, and recreational facilities, are considered as important determinants of smoking and drinking behaviors [15]. In addition, social norms, psychosocial distress, exposure to tobacco and alcohol advertising and their outlets have been proposed as factors at the neighborhood level that may influence tobacco and alcohol use [14,16,17,18]. However, few studies have explored the physical environments of the neighborhoods (e.g., quality, facilities, problems, and walkability) or residents’ perceptions of the physical environment as influences on smoking and alcohol use [4,19]. 

Despite increasing interest in the perceived physical environment of neighborhoods, most studies tend to focus on its relationships with mental health indicators [4,6,20,21] and physical activity [5,8,22,23]. It has been demonstrated that perceptions of neighborhood problems (e.g., poor quality of facilities, less safety) are associated with lower quality of life, greater depressive symptoms and lower physical activity [24]. However, it is unclear which perceived aspects of neighborhoods might be related to smoking and drinking behaviors. The current study examined the relationships between neighborhood environment perceptions and the likelihood of tobacco or alcohol use and binge drinking using individual data on neighborhood perceptions collected from a large sample of adults residing in randomly selected Census block groups in a Midwestern metropolitan area.

## 2. Methods

### 2.1. Sample and Setting

The current study was part of the KC BEST study, a cross-sectional population-based survey on the built environment and health that was conducted in a large Midwestern metropolitan area [25]. The primary aim of KCBEST was to examine how neighborhood environments influenced obesity risk. 

In the KCBEST study, 21 census block-groups were randomly selected within strata based on median yearly family household income and the proportion of minorities in the block groups according to the 2010 U.S. Census. Family household income within a census block group was divided into tertiles (from the population distribution of the household income variable); low, middle, and high income to ensure maximum income differences for the census block groups. Income tertiles were defined as $4999.00 to $23,386.33 for low income; $23,386.34 to $35,569.00 for middle income; and $35,569.01 to $150,001.00 for high income. Seven census blocks were randomly selected from each group. We sampled census blocks with low and high minority representation with a minimum of 19% ethnic minority residents to ensure adequate diversity (*i.e.*, so that no areas that were composed of only one ethnic/racial group were selected). 

To collect individual-level data, households within the selected block groups were solicited until the target number of interviews were reached. Door-to door household interviews were completed with at least 25 household per block group, for a total of 586 participants (30.4% of 1928 adults contacted and eligible). Individuals were eligible to participate if they were between 18 and 74 years of age, had lived in the area at least 12 months, were able to read and understand surveys in English, and were primarily responsible for making food decisions for the household. Pregnant women and individuals who currently had any chronic health conditions or a disability that prevented them from participating in physical activity were excluded from participation. Prior to the interview, eligible participants were informed by a letter that outlined the purpose of the study and requested cooperation. Letters in advance of a household interview have been found to significantly decrease interview refusals [26]. 

Trained interviewers collected data during 60-min face-to-face interviews. Interviewers were instructed to speak to the adult responsible for food decisions for the household. The reason for asking for the person who made food decisions for the household was because the main study (KC BEST) focused on food preparation and selection. The study was approved by the relevant institutional IRBs and informed consent was obtained from all eligible participants. 

### 2.2. Measures

#### 2.2.1. Primary Variables of Interest

*Perceived Neighborhood Characteristics.* The Environmental Module of the International Physical Activity Prevalence Study questionnaires (IPAQ E-module) [27,28] was used to assess perceived neighborhood environments. The IPAQ E-module has 17 environmental items designed for assessing whether neighborhoods are perceived as conducive to physical activity. In this study, we used 10 items (seven core, two recommended, and one optional item) [28] which included questions asking about: (1) residential density; (2) access to destinations; (3) neighborhood infrastructure; (4) aesthetic qualities; and (5) neighborhood safety. These items have been shown to have good reliability and validity [22,27,29,30]. 

*Smoking and Alcohol Use Patterns.* Cigarette smoking and alcohol use were determined using validated questions from the Centers for Disease Control and Prevention (CDC) Behavioral Risk Factor Surveillance System [31]. The smoking questions included: (1) Have you ever smoked at least 100 cigarettes or the equivalent amount of tobacco in your lifetime? (2) Have you ever smoked daily? (3) Do you now smoke daily, occasionally, or not at all? and (4) On the average, how many cigarettes do you now smoke a day? Those who answered daily or occasionally were considered to be smokers and those who answered not at all were considered to be non-smokers (including former smokers). Heavy drinking was defined as consuming more than one and two drinks of any alcohol per day for women and men respectively (http://www.cdc.gov/alcohol/faqs.htm). In addition, binge drinking was determined by whether they consumed five or more drinks of alcohol on an occasion during the past 30 days [32]. 

#### 2.2.2. Secondary Variables

*Health Characteristics.* Body Mass Index (BMI) was calculated as weight (kg) divided by height squared (m^2^). Physical activity level was determined using the International Physical Activity Questionnaire (IPAQ; Guidelines for data processing and analysis of the IPAQ—Short and Long forms. www.ipaq.ki.se/scoring.pdf, November 2005), which asked participants to report their activities spent in physical activity for at least 10 min during the last 7 days. IPAQ scores were estimated by weighting time spent in each activity intensity with its estimated metabolic equivalent (MET) energy expenditure. Participants’ physical activity levels were categorized as low (<600 MET-min/week), moderate (600–3000 MET-min/week), and high (>3000 MET-min/week). Participants also were asked to provide the number of poor physical health days experienced during the last 30 days [33]. This question has established validity and reliability, is predictive of longitudinal health outcomes, and is used as part of an overall health rating system for the U.S. [33,34].

*Sociodemographic Characteristics.* Participants’ sociodemographic characteristics included age, gender, marital status, race/ethnicity, education, employment status, and annual household income 

*Psychosocial Characteristics.* Depressive symptoms were measured with the 10-item version of the Center for Epidemiologic Studies Depression scale (CES-D 10) [35]. It was found to have comparable reliability estimates to those reported for the original CES-D and had strong internal consistency (Chronbach’s α = 0.9) and test-retest reliability (*r* = 0.8) [35,36]. In addition, the 4-item version of the Perceived Stress Scale (PSS) [37] was employed to assess the degree to which participants often felt or thought the way described by the items in the past month. The PSS has been found to be highly reliable in the general U.S. population [37,38]. For both scales, higher scores indicated higher levels of psychological distress.

*Neighborhood SES Indicators.* Neighborhood demographic variables collected included median yearly family household income and percent of the minority population within a census block group. As explained previously, the selected block groups were divided into three categories based on their median yearly family household income; lowest, middle, and highest tertiles; and were median-split into 2 categories based on their minority population; lowest and highest percentage of minority. 

### 2.3. Analytic Plan

*Factor Analysis.* The factorability of the perceived environment covariance matrix was examined using Dziuban and Shirkey’s test [39]. The overall Kaiser-Meyer-Olkin (KMO) measure of sampling adequacy was 0.71, which places it in the “average” range. The Bartlett sphericity test was significant (*p* < 0.001) and the determinant of the matrix was in the acceptable range. Thus, the perceived environment covariance matrix appeared appropriate for factor analysis. In order to condense the perceived environment variables from the IPAQ-E module, principal component analysis with varimax rotation using SAS PROC FACTOR was used to identify groups of related perceived neighborhood attributes. Component loadings ≥0.4 were considered significant [40]. The analysis identified three components accounting for 59.1% of the variance. All three dimensions demonstrated acceptable reliability: (1) Neighborhood’s Infrastructures (4 items); (2) Neighborhood Safety (2 items); and (3) Neighborhood Accessibility (2 items). Therefore, these dimensions were used for further analysis. 

The Neighborhood’s Infrastructures dimension included: (1) “The sidewalks in my neighborhood are well maintained and not obstructed”; (2) “There are sidewalks on most of the streets in my neighborhood”; (3) “There are facilities to bicycle in or near my neighborhood” and (4) “My neighborhood has several free or low cost recreation facilities”. The Neighborhood Safety dimension included: (1) “The crime rate in my neighborhood makes it unsafe to go on walks at night”; and (2) “There is so much traffic on the streets that it makes it difficult or unpleasant to walk in my neighborhood”. The Neighborhood Accessibility dimension included: (1) “Many shops, stores, markets or other places to buy things I need are within easy walking distance of my home”; (2) It is within a 10–15 min walk to a transit stop from my home; and (3) “There are many interesting things to look at while walking in my neighborhood”. 

Internal consistency for each of the dimensions was demonstrated to be adequate using Cronbach’s Alpha; 0.70, 0.60, and 0.60 for Neighborhood’s Infrastructures, Safety, and Accessibility dimensions, respectively. A score for each dimension was constructed by summing responses to each item included; higher scores indicated better ratings of the perceived neighborhood environments. For the bivariate analyses, each category was dichotomized on the basis of a median split, dividing the index into negative (*i.e.*, unattractive or unsafe neighborhood) and positive (*i.e.*, attractive or safe neighborhood) overall perception. 

*Data Analysis.* We hypothesized that how people perceived their neighborhood environment (positive/negative) would be related to tobacco use and alcohol consumption. Thus, our models focused on the relationships between neighborhood perception variables and tobacco and alcohol use. Potential confounding variables that have been identified in the literature or have been thought to be conceptually related to alcohol and tobacco use or neighborhood perceptions also were included in all models (e.g., age in 5-year intervals, other demographic factors, physical activity level, BMI, and self-reported health). Independent samples t-tests were used to compare gender differences on continuous variables, while Chi-Square analysis was used to compare gender differences on categorical variables. The literature suggests that neighborhood characteristics are differently perceived and observed between men and women, so the data were analyzed separately. To examine the likelihood of being a current smoker, heavy drinker, and/or a binge drinker, mixed models were created within the SAS PROC GLIMMIX (SAS 9.2, SAS Institute Inc., Cary, NC, USA) with neighborhood as a random effect in each model to adjust for the sampling approach used in the study. Statistical models produced odds ratios (ORs) which represent the odds of having the outcome of interest (being a smoker, binge and heavy drinker). 

## 3. Results

Table 1 provides the sociodemographic, neighborhood, and psychosocial characteristics and health behaviors of the study population. 

**Table 1:** Participants sociodemographic, neighborhood, psychosocial characteristics and health behaviors (*n* = 586) **^a^**.

Characteristics	All	Women (*n* = 409)	Men (*n* = 177)
**Sociodemographic Characteristics**			
Age (years)	45.0 ± 14.7	45.0 ± 14.3	45.1 ± 15.7
Marital Status, %			
Single	42.9	40.9	47.4
Married	57.1	59.1	52.6
Race, %			
White, non-Hispanic	63.1	62.6	64.4
Racial/Ethnic Minority	36.9	37.4	35.5
Education			
≤12 years	25.8	25.69	26.01
>12 years	74.2	74.31	73.99
Employment Status, %			
Unemployed	40.1	42.5	34.7
Employed	59.9	57.5	65.3
Annual HH income	37,346.5 ± 55,708.6	**33,825.4 ± 48,733.9**	**45,482.8 ± 68,661.2**
**Perceived Neighborhood Characteristics**			
Infrastructures	10.7 ± 3.5	10.5 ± 3.6	10.9 ± 3.4
Safety	6.1 ± 1.7	**6.0 ± 1.8**	**6.5 ± 1.5**
Accessibility	7.8 ± 2.4	**7.6 ± 2.4**	**8.1 ± 2.4**
**Neighborhood SES Indicators**			
Block Group Median Income, %			
Lowest tertile	35.4	33.17	40.57
Middle tertile	30.4	30.47	30.29
Highest tertile	34.2	36.36	29.14
Block Group Minority Population, %			
Lowest	52.2	49.88	57.71
Highest	47.8	50.12	42.29
**Psychosocial Characteristics**			
Depression	2.2 ± 2.2	**2.3 ± 2.2**	**1.9 ± 1.9**
Perceived Stress	3.9 ± 3.0	3.9 ± 3.1	3.9 ± 2.8
**Health Characteristics**			
Smoker, %	25.4	23.56	29.48
Heavy drinkers, %	59.2	61.12	54.80
Binge drinkers, %	12.7	**8.26**	**21.09**
Physical Activity, % low	14.5	14.2	15.3
Number of self-reported physical health days	2.7 ± 5.5	2.7 ± 5.7	2.5 ± 4.9
BMI, kg/m^2^	28.4 ± 7.6	28.3 ± 8.0	28.6 ± 6.6

**^a^** mean ± SD unless noted otherwise; Bolded values indicate statistically significant differences between gender based on *t*-test and Chi-Square values, *p* < 0.05.

Overall, the mean age of the sample population was 45 years and 69.8% were female. The majority of the sample were non-Hispanic Whites who were employed and completed high school. There were no significant sociodemographic differences between men and women except for annual household income. With respect to psychosocial and health characteristics, female participants were more likely to report having depressive symptoms, but less likely to be binge drinkers in the past 30 days compared to male respondents. Male participants had significantly higher scores on the measures of perceived neighborhood safety and neighborhood accessibility than their female counterparts (*p* < 0.05). 

Table 2 presents ORs and 95% confidence intervals for the associations between individual and perceived neighborhood characteristics and the likelihood of being current smokers, or heavy and binge drinkers. Mixed model analyses were used to examine variables that influenced smoking or problem drinking patterns. Statistically significant associations between neighborhood perceptions and the likelihood of smoking or binge drinking were only found among male participants. Men who had positive perceptions of neighborhood infrastructure were almost five times less likely to smoke (OR = 4.8; *p* < 0.05), and eight time less likely to binge drink (OR = 7.7; *p* < 0.05). However, male participants who had positive perceptions on neighborhood accessibility were almost seven time more likely to smoke (OR = 6.7; *p* < 0.05). The only health characteristic that was significantly associated with the smoking was physical activity level. Men who had medium or high level of physical activity were five times more likely to smoke compared to those who had low level of physical activity. 

**Table 2:** Logistic regression models of smoking, heavy drinking and binge drinking prevalence.

Characteristics	Smoking		Heavy Drinking		Binge Drinking	
	Women OR (95% CI)	Men OR (95% CI)	Women OR (95% CI)	Men OR (95% CI)	Women OR (95% CI)	Men OR (95% CI)
**Perceived Neighborhood Characteristics **						
Infrastructures						
Negative Perceptions (ref)	1.0	1.0	1.0	1.0	1.0	1.0
Positive Perceptions	1.2 (0.7−2.2)	**0.2 (0.1−0.8)**	1.2 (0.7−2.1)	0.8 (0.3−1.9)	1.9 (0.6−6.7)	**0.1 (0.1−0.7)**
Safety						
Negative Perceptions (ref)	1.0	1.0	1.0	1.0	1.0	1.0
Positive Perceptions	0.9 (0.5−1.6)	3.3 (0.7−15.8)	1.4 (0.8−2.4)	0.8 (0.3−2.5)	2.2 (0.6−8.8)	1.4 (0.2−8.4)
Accessibility						
Negative Perceptions (ref)	1.0	1.0	1.0	1.0	1.0	1.0
Positive Perceptions	1.3 (0.7−2.4)	**6.7 (1.5−30.6)**	1.4 (0.9−2.4)	1.2 (0.5−3.0)	1.2 (0.4−4.4)	1.2 (0.2−6.1)
**Health characteristics**						
BMI (kg/m^2^)	1.0 (0.9−1.0)	0.9 (0.8−1.0)	0.9 (0.9−1.2)	0.9 (0.9−1.0)	1.0 (0.9−1.1)	0.9 (0.8−1.1)
Physical Activity						
Low (ref)	1.00	1.0	1.0	1.0	1.0	1.0
Medium/High	1.3 (0.8−2.1)	**5.2 (1.5−18.4)**	1.0 (0.9−1.4)	1.4 (0.7−2.7)	1.7 (0.6−4.8)	3.0 (0.5−17.7)
Self-reported Health	1.0 (0.9−1.1)	1.2 (0.9−1.4)	1.0 (0.9−1.0)	1.0 (0.9−1.1)	1.0 (0.9−1.1)	1.2 (0.9−1.5)
**Sociodemographic Characteristics**						
Age (10-year interval)	**0.8 (0.6−0.9)**	0.7 (0.4−1.2)	**0.7 (0.6−0.9)**	0.9 (0.6−1.2)	0.8 (0.5−1.4)	0.7 (0.4−1.4)
Marital Status						
Single (ref)	1.0	1.0	1.0	1.0	1.0	1.0
Married	0.7 (0.4−1.2)	0.6 (0.2−2.5)	0.6 (0.3−1.1)	0.8 (0.3−2.2)	0.3 (0.1−1.2)	0.8 (0.2−4.3)
Race						
White, non-Hispanic (ref)	1.0	1.0	1.0	1.0	1.0	1.0
Minorities	0.7 (0.3−1.4)	0.2 (0.04−1.1)	**0.5 (0.3−0.9)**	0.6 (0.2−1.7)	2.2 (0.6−7.9)	1.1 (0.12−9.30)
Education						
≤12 years (ref)	1.0	1.00	1.0	1.0	1.0	1.0
>12 years	0.7 (0.4−1.3)	1.00 (0.2−5.9)	1.0 (0.5−1.8)	0.5 (0.2−1.7)	1.1 (0.3−4.8)	0.4 (0.1−2.6)
Employment Status, %						
Unemployed (ref)	1.0	1.0	1.0	1.0	1.0	1.0
Employed	1.0 (0.6−1.8)	0.6 (0.2−2.7)	1.5 (0.9−2.6)	2.4 (0.9−6.5)	0.5 (0.1−1.7)	1.4 (0.2−9.7)
**Psychosocial Characteristics**						
Depression	1.1 (0.9−1.2)	**1.8 (1.2−2.7)**	0.9 (0.8−1.1)	0.8 (0.6−1.1)	1.0 (0.7−1.4)	1.3 (0.7−2.1)
Perceived Stress	1.0 (0.9−1.2)	0.8 (0.6−1.0)	1.0 (0.9−1.2)	1.1 (0.9−1.3)	1.1 (0.8−1.3)	0.9 (0.6−1.4)

Bolded values indicate statistically significant differences. CI: Confident interval. Results are adjusted for neighborhood SES and percentage of minority in block group using a multilevel modeling approach.

Depression also showed a significant relationship with smoking in male respondents (OR = 1.8; *p* < 0.01). Among female participants, being younger was associated with smoking (OR = 1.3; *p* < 0.05) and heavy drinking (OR = 1.4; *p* < 0.05), while heavy drinkers were more likely to be White, non-Hispanics (OR = 2.0; *p* < 0.05). 

## 4. Discussion

We have explored the relationship between perceived neighborhood environments and individual sociodemographic and psychosocial characteristics on the likelihood of being a current smoker, heavy and/or binge drinker. We found that perceived neighborhood problems were strongly associated with the likelihood of current smoking and binge drinking in men. Male respondents who had negative perceptions of neighborhood infrastructures were more than four times more likely to be current smokers and eight times more likely to be binge drinkers, which is consistent with results from a previous study assessing perceived neighborhood problems and smoking and drinking [41]. In contrast, men who had negative perceptions of neighborhood accessibility were less likely to smoke. This finding is in line with several studies reporting that convenient accessibility to shops or stores has been liked with smoking [16,42]. Chuang [16] suggested that greater access to stores may accentuate greater access to tobacco products and more pro-tobacco influences. In addition, Pearce *et al.* [42] hypothesized that, where shops, supermarkets, and stores are more accessible, the opportunities of tobacco consumption are increasing. However, similar relationships between those health behaviors and neighborhood perceptions were not found among female participants. These results might be because men and women perceive neighborhood environment characteristics differently, which could influence on how they respond to the neighborhood situation. For example, several studies have found that females who had lower perceptions of neighborhood infrastructure or safety were more likely to constrain their health-risk activity compared to their male counterparts [43,44,45]. One possible explanation for this difference might be related to gender roles in that women tend to be the main caregivers and role model for children which may lead them to make healthier choices [46]. Unlike previous work demonstrating and association between perceived neighborhood safety and smoking and alcohol consumption [41,47], this study failed to replicate such relationships.

Depressive symptoms increased the likelihood of smoking among men, suggesting that those who experience distress might use tobacco as a coping strategy or distress reliever [48,49]. In addition, negative neighborhood perceptions have been found to be associated with mental health problems through the effects of psychological stress [50,51] and those who were in those conditions might use cigarettes and alcohol to reduce stress, distress and to escape problems [14,52]. Moreover, aspects and physical features of neighborhoods (*i.e.*, poor-quality housing, absence of basic resources, lack of health care or recreational facilities, lack of access to public transportation), and density of buildings may function as stressors that could influence smoking and alcohol consumption [15,53].

Several sociodemographic variables were found to be associated with health behaviors. In accordance with previous research, younger women were more likely to smoke, and use alcohol excessively (*i.e.*, be heavy drinkers) when compared to their older counterparts [54,55]. In addition, female participants who identified as non-Hispanic White were more likely to be heavy drinkers than were women of color [56]. O’Hare [57] implied that this drinking pattern could be mediated by cultural factors and social learning, which has been supported by several other studies [56,58,59]. Thus ethnic/race drinking differences should be further explored. 

Physical activity level was found to have a positive association with being a current smoker. Men who had medium and high physical level tended to smoke more than those who were physically inactive, which is in opposition to findings from studies that reported a negative association [60]. However, our findings are in agreement with those of others who reported significant associations between smoking and physical activity [61,62,63]. The possible explanation might be due to a compensation effect [63,64], in which smokers tended to exercise more in order to compensate for the negative health effect of smoking. 

Several limitations about this study should be acknowledged. Due to the cross-sectional nature of this study, the associations between neighborhood perceptions and health behaviors could be influenced by other factors such as personality, beliefs or attitudes which may cause individuals to choose to live or behave in a particular way. For example, people tend to choose their neighborhoods based on similar lifestyle profile of neighbors [65] or people who always engage in risky behaviors would express similar attitudes regardless of their neighborhoods [66]. Longitudinal studies examining these relationships are needed and would allow us to better understand the direction of the relationships between exposures and outcomes. In addition, some of the associations demonstrated wide 95% confidence intervals, indicating that the effect of these variables are uncertain and might be due to the small sample size and further information is needed. Despite these limitations, our findings suggest that some neighborhood perception variables are associated with tobacco and alcohol use among men in our sample and that this association could be influenced by stress and depression presented in the environments [24]. Therefore, it is significant to identify the mechanism that may explain these relationships. 

## 5. Conclusions

Our findings indicate that the perceived neighborhood environment, especially neighborhood infrastructures, was predictive of health behaviors among participants in this sample, even after adjusting for key confounders. Interestingly, other individual characteristics such as demographic variables and psychosocial variables also demonstrated strong relationship with those health outcomes. Thus, closer attention may need to be paid to the role of neighborhood environment characteristics along with individual-level characteristics in influencing unhealthy behaviors. 
